# Effects of species traits and environmental predictors on performance and transferability of ecological niche models

**DOI:** 10.1038/s41598-019-40766-5

**Published:** 2019-03-12

**Authors:** Adrián Regos, Laura Gagne, Domingo Alcaraz-Segura, João P. Honrado, Jesús Domínguez

**Affiliations:** 1 Departamento de Zooloxía, Xenética e Antropoloxía Física, Universidade de Santiago de Compostela, Santiago de Compostela, Spain; 2 Research Center in Biodiversity and Genetic Resources (CIBIO/InBIO), Universidade do Porto, Vairão, Portugal; 3 Universitè de Niza Sophia Antipolis, Nice, France; 4 Department of Botany and Inter-Universitary Institute for Earth System Research, University of Granada, Granada, Spain; 5 Andalusian Center for the Assessment and Monitoring of Global Change (CAESCG), University of Almería, Almería, Spain; 6 Faculdade de Ciências, Universidade do Porto, Porto, Portugal

## Abstract

The ability of ecological niche models (ENMs) to produce robust predictions for different time frames (i.e. temporal transferability) may be hindered by a lack of ecologically relevant predictors. Model performance may also be affected by species traits, which may reflect different responses to processes controlling species distribution. In this study, we tested four primary hypotheses involving the role of species traits and environmental predictors in ENM performance and transferability. We compared the predictive accuracy of ENMs based upon (1) climate, (2) land-use/cover (LULC) and (3) ecosystem functional attributes (EFAs), and (4) the combination of these factors for 27 bird species within and beyond the time frame of model calibration. The combination of these factors significantly increased both model performance and transferability, highlighting the need to integrate climate, LULC and EFAs to improve biodiversity projections. However, the overall model transferability was low (being only acceptable for less than 25% of species), even under a hierarchical modelling approach, which calls for great caution in the use of ENMs to predict bird distributions under global change scenarios. Our findings also indicate that positive effects of species traits on predictive accuracy within model calibration are not necessarily translated into higher temporal transferability.

## Introduction

Ecological niche models (ENMs) based on correlative species-environment relationships are widely used to assess the impact of past and future global change on biodiversity^1,2^. Despite the long-standing and important role of ENMs in global change research^3^, these correlative approaches present important shortcomings that challenge their applicability in a changing, highly dynamic world (see e.g.^4–6^). One such limitation is the low transferability (i.e. model extrapolation) of parameterised models to other regions or times beyond the range of data used for model fitting (see^7^, for a review). However, the spatial and temporal transferability of ENMs is rarely evaluated prior to predicting species distribution in different regions or times.

Previous studies have provided evidence that model transferability is influenced by the modelling technique, as different algorithms show different sensitivities to spatial dimensionality and correlation^8–12^. Ensemble modelling and consensus methods have been proposed as an alternative to avoid overreliance on a single technique and reduce uncertainty across individual models^13^. Moreover, excessively complex models risk overfitting the data used for calibration, ultimately leading to predictions that can be too specific to the reference system to be transferable^14^. Most parsimonious models – built upon a small set of predictors – are expected to lead to greater transferabilities^15^. Thus, one critical step to ensure that models are ecologically meaningful, and therefore may support transferability^14^, is the choice of predictor variables^16,17^. However, many studies appear to use only environmental data that are readily available, while failing to consider other variables that may be ecologically relevant to the species distribution (i.e. causal rather than substitute predictors)^18^, and often missing important environmental drivers. New research on how the integration of key environmental drivers affects the transferability of ENMs is urgently needed given the increasing consensus on the importance of addressing the combined impacts of future climate and land-use/cover changes on biodiversity^19–21^. In this regard, the incorporation of less common but equally important predictors is now increasingly feasible, as remote sensing data are more readily available^22^. This broad range of available predictors can help improve the performance of ENMs and, in turn, their transferability^14,23,24^. This is the case for the intra- and inter-annual variability and seasonal dynamics of vegetation indices obtained from monthly images captured by satellite sensors. Several metrics summarising such dynamics are considered robust, integrative descriptors of ecosystem functioning^25,26^ and have recently been found to be meaningful predictors for species distributions and their temporal dynamics^27–29^. However, to the best of our knowledge, there is only one study that has assessed the role of these remotely-sensed ecosystem functional variables on model transferability^30^, although without considering the potential advantages of combining those variables with other relevant environmental drivers such as climate and land use change.

To advance on the application of model extrapolation in time and space, a better understanding of the processes and conditions that affect transferability is needed^14^. The ecology of species has also been demonstrated to impact model performance^31–33^ and predictor ranking^34^. Indeed, the effects of species traits (i.e., characteristics or qualities of the organisms of a species influencing performance or fitness)^35^ on the predictive ability of ENMs were found to override any differences in modelling technique, as these traits may reflect the different responses of species to processes that control species distributions^36^. For instance, a global meta-analysis has showed that species traits such as body size, dispersal model and trophic position may be good indicators of predictability^37^. Other studies found that non-endemic species with greater dispersal capacity, intermediate levels of prevalence, and little fire adaptation held higher model transferability than endemic species with limited dispersal capacity^38^. In addition, ENMs based upon climate, land cover and soil type variables performed less well for highly mobile species with large ranges^39^. A recent study also highlighted that the predictive ability of ENMs differs with regard to life history characteristics such as range, migration, habitat and rarity of a species^40^.

Despite the growing body of research and evidence, the relationship between specific traits, predictor variables and model transferability still remains poorly understood^7,14^. To address this caveat, here we tested four primary hypotheses involving the role of species traits and environmental predictors in model performance and transferability (see Table 1 for a detailed description):

**Table 1:** Hypotheses and sub-hypotheses, expectations arising from each hypothesis, and corresponding comparisons of model accuracy.

Hypothesis	Sub-hypothesis	Expectations and rationality	Comparison
(H1) Model transferability hypothesis		Model accuracy usually evaluated with a split-sample approach, i.e. repeatedly and randomly leaving out a subset of data used for calibration (‘Cross-validation’), will be higher within than beyond the model calibration time frame^7,103^. We also expect higher model accuracy after testing the temporal projections against temporally (but not spatially) independent data (‘Internal TT’) than against spatially and temporally independent data (‘External TT’). This hypothesis aims to assess if cross-validation procedures are good indicators of model transferability.	‘Crossvalidation’ vs ‘Internal TT’ and ‘External TT’.
(H2) Environmental-predictor hypothesis		The lack of ecologically relevant predictors will substantially reduce ENM performance and transferability^6,7^. While holding the number of variables constant (i.e., trivariate models), model accuracy − both within and beyond the model calibration time period − will increase with the combination of different types of environmental predictors related to climate, habitat availability, and ecosystem functioning conditions.	‘Individual’ vs ‘combined’ models.
(H3) Hierarchical-integration hypothesis		Model accuracy will improve, particularly beyond the model calibration time period, with the hierarchical integration of environmental drivers whose effects are more evident and relevant at regional scales (i.e., climate) with those more important at local scales (i.e., land use/cover and ecosystem functioning)^41–43^. Climate-driven models calibrated at regional scales (i.e., Iberian Peninsula) will be able to capture a larger range of species distributions and, therefore, wider range of climate conditions where the species can currently occur or not^104^. These ‘regional’ climate-driven models will be able to define the climate niche of species more generally (i.e. fewer omission errors) than ‘local’ models, and they can then be improved by the incorporation of land use/cover and ecosystem functioning at finer scales (i.e. Gerês-Xurés Biosphere Reserve).	‘Hierarchical’ vs ‘non-hierarchical’ modelling approaches.
(H4) Species-traits hypothesis		Performance and transferability of ENMs will be influenced by species traits^31,38,40,44^. Species ecological characteristics will affect our ability to describe their distributions within the time frame used for model fitting (i.e. calibration) and to predict distributional shifts through time (i.e. temporal projections). If so, species’ traits might help to identify environmental predictors more suitable for predicting species’ distribution.	Species traits across crossvalidation, ‘TT Internal’ and ‘TT external’.
	**(H4.1). Biogeographic-climate hypothesis**	If climate is the determining factor of bird species distributions (*sensu*)^105,106^, the performance of ENMs based upon climate variables will be strongly dependent on the species traits related to their biogeographic origin. Given that Mediterranean species are relatively scarce and rare in our study area, we can expect that their distributions can be *a priori* more easily predictable in space and time (due to combined effects of a narrow niche and small range size)^97^ than for Eurosiberian species, yielding the highest model accuracy.	‘Eurosiberian’ vs ‘Mediterranean’
	**(H4.2). Habitat-landcover hypothesis**	Model performance and transferability will be higher for habitat-specialists than for generalists when predicted with models built with land cover (but not necessarily with climate) variables^95,96^. However, the predictive accuracy of our models should not be affected by species’ habitat preference (i.e. forest or open habitat).	‘Habitat-specialist’ vs ‘habitat-generalist’ for ENMs based upon land cover, climate variables; and, ‘open-habitat’ vs ‘forest-habitat’ for ENMs based upon land cover, climate variables.
	**(H4.3) Phenology-EFAs hypothesis**	Migrating birds track vegetation dynamics; i.e., the birds move with the seasonally progressing green-up of vegetation^28^, so species traits related to their phenology will potentially contribute to explain the performance and transferability of ENMs based upon ecosystem functioning attributes (‘EFAs’)^26^, which are derived from inter-annual variability and seasonal dynamics of vegetation indices.	‘Migrant’ vs ‘sedentary’ for ENMs based upon EFA
	**(H4.4) Trait-predictor hypothesis**	Since EFAs are directly affected by climate and land use/cover change^25^, they can potentially provide information about the integrative response of species to global environmental change^102^, Thus, we would expect that those species traits with impacts on the performance and transferability of climate and/or land-cover models will also affect our predictions based upon EFAs.	Species traits for ENMs based upon EFAs vs ENMs based upon climate and/or land-cover variables.

(H1) **Model transferability hypothesis**: Model accuracy will be higher within than beyond the model calibration time frame^7^.

(H2) **Environmental-predictor hypothesis**: Model accuracy, both within and beyond the model calibration time period, will increase with the combination of different types of relevant environmental predictors related to climate, habitat availability and ecosystem functioning conditions^6,7^.

(H3) **Hierarchical-integration hypothesis**: Model accuracy will improve, particularly beyond the model calibration time period, by hierarchically integrating environmental drivers whose effects are more evident and relevant at regional scales (i.e. climate) with those found to be more important at local scales (i.e. land use/cover and ecosystem functioning)^41–43^.

(H4) **Species-traits hypothesis**: Model performance and transferability will be influenced by species traits^31,38,40,44^ (see Table 1 for expectations and sub-hypotheses).

To test these hypotheses, we compared the predictive accuracy of ENMs based upon (1) climate, (2) land-use/cover and (3) ecosystem functional attributes, and (4) the combination of these factors, for 27 bird species within the model calibration period (year 2000) and in a different time frame (year 2010), in a highly dynamic landscape of NW Iberia (Gerês-Xurés Biosphere Reserve). The predictive accuracy was aggregated by species traits related to biogeographic origin, migratory status and habitat preference and specialization.

## Methods

### Study site

**Figure 1:**
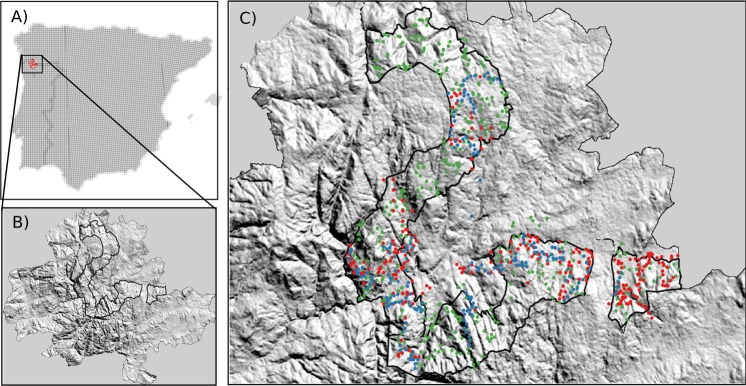
Location of the study area (Gerês-Xurés Mountains) in the Iberian Peninsula. (**A**) 10-km squares in the Iberian Peninsula. (**B**) Location of the study area in the Gerês-Xurés transboundary UNESCO Biosphere Reserve. (**C**) Spatial distribution of the point counts used for sampling bird communities: 5-min point counts for 2000 (i.e. calibration dataset) (red dots), 5-min point counts for 2010 (i.e. ‘Internal TT’ dataset) (blue dots), and 20-min point counts for 2010 (i.e. ‘External TT’ dataset) (green dots). Maps generated with the QGIS 2.16.2 https://www.qgis.org/es/site/.

The study area is the Baixa Limia-Serra do Xurés Natural Park (*c*. 29 345 ha), located in the northwest of the Iberian Peninsula (Fig. 1A). It is included in the Gerês-Xurés transboundary UNESCO Biosphere Reserve (Fig. 1B) and protected under EU legislation (Habitat and Bird Directives). The zone is a mountain range, with elevation gradient between 323 and 1529 m a.s.l., located in the transition between the Mediterranean and Eurosiberian biogeographic regions and in the proximity of the Atlantic coast^45^, making it particularly susceptible to climate change. The landscape is mainly dominated by shrubland (69%) and forest (21%)^46^. The area has been greatly affected by the abandonment of traditional agricultural and livestock activities, which has favoured vegetation encroachment and recent forest expansion^46,47^. The area is also subjected to a high frequency of human-caused fires − linked to long-standing socio-economic difficulties in rural communities − and to a fast recovery of vegetation after fire^48^, resulting in an unstable and highly dynamic system^46^.

### Bird data

We used occurrence (presence/absence) data for bird species at two different scales (both spatial extent and resolution): (1) Iberian extent at 10-km resolution and (2) Gerês-Xurés Mountains at 230-m resolution (Fig. 1). At the Iberian scale, bird data were obtained from the combination of the Second Spanish and Portuguese Atlases of Breeding Birds^49,50^. This dataset documents the occurrence of breeding bird species in 6212 grid cells of 10-km resolution (Fig. 1A), with field data mostly collected between 1998 and 2005. At the Gerês-Xurés scale, bird data was surveyed in 2000 and 2010 using two different sampling methodologies. A set of 344 5-min point counts with unlimited distance were carried out in 2000 (hereafter: ‘calibration dataset’) (Fig. 1C). This initial dataset was partly replicated in 2010 by re-sampling a subset of 204 5-min point counts to evaluate the temporal transferability of ENMs (i.e. internal temporal transferability assessment; hereafter: ‘Internal TT’ dataset). Simultaneously, another set of 384 20-min point counts with a limited distance of 80 m was surveyed twice during the spring of 2010 to form a spatially and temporally independent data set (i.e. external temporal transferability assessment; hereafter: ‘External TT’ dataset) (Fig. 1C). The censuses were undertaken during the breeding season (May–June). To avoid possible detection biases caused by the time of survey, wind speed or cloud cover, all censuses were carried out during the 4 h after sunrise (peak vocal activity) and under uniform weather conditions (days without marked rainfall or wind). In the ‘External TT’ survey, the point counts were resampled in the afternoon during the 4 h before sunset (peak vocal activity).

From the initial set of species, we only selected those with more than 15 presences to ensure a minimum of 5 occurrences per predictor (i.e. to avoid overfitting the models)^51^ (see Table 2 for a list of target species). These species were grouped in different guilds based upon their ecological traits related to biogeographical origin (Eurosiberian/Mediterranean), phenology (sedentary/migrant), and habitat specialization (generalist/specialist) and preference (forest/open habitat), information extracted from different sources, from continental to local atlases^49,50,52–55^ (Table 2).

**Table 2:** Traits related to biogeographic origin, habitat preference and specialization, and phenology of each target bird species.

Common name	Scientific name	Acronym	Biogeographic origin	Habitat specialization	Habitat preference	Phenology
Common Woodpigeon	*Columba palumbus*	CPAL	Mediterranean	Generalist	Forest	Sedentary
European Turtle Dove	*Streptopelia turtur*	STUR	Mediterranean	Specialist	Forest	Migrant
Common Cuckoo	*Cuculus canorus*	CCAN	Eurosiberian	Generalist	Forest	Migrant
European Green Woodpecker	*Picus viridis*	PVIR	Eurosiberian	Generalist	Forest	Sedentary
Eurasian Skylark	*Alauda arvensis*	AARV	Eurosiberian	Specialist	Open habitat	Sedentary
Eurasian wren	*Troglodytes troglodytes*	TTRO	Eurosiberian	Generalist	Forest	Sedentary
Dunnock	*Prunella modularis*	PMOD	Eurosiberian	Generalist	Open habitat	Sedentary
European robin	*Erithacus rubecula*	ERUB	Eurosiberian	Generalist	Forest	Sedentary
Common Stonechat	*Saxicola torquata*	STOR	Eurosiberian	Specialist	Open habitat	Sedentary
Common Blackbird	*Turdus merula*	TMER	Eurosiberian	Generalist	Forest	Sedentary
Dartford Warbler	*Sylvia undata*	SUND	Mediterranean	Specialist	Open habitat	Sedentary
Common Whitethroated	*Sylvia communis*	SCOM	Mediterranean	Specialist	Open habitat	Migrant
Eurasian Blackcap	*Sylvia atricapilla*	SATR	Eurosiberian	Specialist	Forest	Migrant
Iberian Chiffchaff	*Phylloscopus ibericus*	PIBE	Eurosiberian	Specialist	Forest	Migrant
Common Firecrest	*Regulus ignicapilla*	RIGN	Eurosiberian	Specialist	Forest	Sedentary
European crested tit	*Lophophanes cristatus*	PCRI	Eurosiberian	Specialist	Forest	Sedentary
Coal Tit	*Periparus ater*	PATE	Eurosiberian	Specialist	Forest	Sedentary
Great Tit	*Parus major*	PMAJ	Eurosiberian	Specialist	Forest	Sedentary
Short-toed Treecreeper	*Certhia brachydactyla*	CBRA	Eurosiberian	Specialist	Forest	Sedentary
Eurasian Golden Oriole	*Oriolus oriolus*	OORI	Eurosiberian	Specialist	Forest	Migrant
Red-backed shrike	*Lanius collurio*	LCOL	Eurosiberian	Specialist	Open habitat	Migrant
Eurasian jay	*Garrulus glandarius*	GGLA	Eurosiberian	Specialist	Forest	Sedentary
Common Chaffinch	*Fringilla coelebs*	FCOE	Eurosiberian	Generalist	Forest	Sedentary
European Serin	*Serinus serinus*	SSER	Mediterranean	Generalist	Forest	Migrant
European greenfinch	*Carduelis chloris*	CCHL	Mediterranean	Specialist	Forest	Migrant
Common linnet	*Carduelis cannabina*	CCANN	Mediterranean	Specialist	Open habitat	Sedentary
Rock Bunting	*Emberiza cia*	ECIA	Eurosiberian	Specialist	Open habitat	Sedentary

### Environmental data

#### Climate variables

Climate variables were calculated from meteorological stations in Spain and Portugal by using multiple regression techniques (see details in^56^). In particular, 19 climate variables were computed for each year from monthly time series of air temperatures (mean maximum and mean minimum temperature) and total precipitation by using the function ‘*biovars*’ available in the R package ‘*dismo*’, version 1.1–4^57^. From the initial set of 19 climate variables, we selected the two most ecologically relevant for the bird species according to expert knowledge and scientific literature^58,59^: (1) the maximum temperature of the warmest month, and (2) the average annual precipitation. A third predictor variable − (3) seasonality of precipitation (i.e. coefficient of variation) − was also selected after ensuring no problems related to collinearity with the other climate variables (see Supplementary Information S1 for correlations between pairs of predictors, description and visualization). Climate variables were resampled from their original resolution of 200 m to 230 m in order to match the spatial resolution of the EFAs.

#### Land-use/cover variables

The land-use/cover variables were derived from optical and thermal multispectral bands of Landsat TM and ETM + images acquired over the same temporal sequence as the bird sampling was carried out (20 March 2000, 8 June 2000, 24 June 2000, 19 May 2010 and 30 July 2010). The land-cover classification was obtained using a hybrid classification procedure, which combines unsupervised and supervised strategies^47^ (see details in Supplementary Information S2). Land-use/cover types were then aggregated in broad categories to describe the landscape composition, and three main categories were selected to describe habitat preference for the target bird species: (1) scrubland, (2) forest and (3) cropland. Land-use/cover variables represent the percentage of these three main categories in each geographic unit (i.e. grid cell), and they were resampled from their original resolution of 30 m to the spatial resolution of the EFAs (~230 m).

#### Ecosystem functioning variables

Ecosystem functional attributes (EFAs) were derived from the Enhanced Vegetation Index (EVI) obtained from 16-day maximum value composite images captured by the Moderate Resolution Imaging Spectroradiometer (MODIS) sensor at a spatial resolution of ∼230 m. EVI is related to the fraction of photosynthetically active radiation intercepted by vegetation. EVI proved to have better sensitivity in high biomass regions and lower influence from the canopy background and atmospheric noise than other vegetation indices^60^. Three independent metrics of the EVI seasonal dynamics were calculated for 2001 (MODIS complete year closest to 2000) and 2010: (1) EVI annual mean, an estimator of annual primary production, which is one of the most integrative descriptors of ecosystem functioning (productivity indicator); (2) EVI seasonal standard deviation, a descriptor of the difference in carbon gains between seasons (seasonality indicator); and (3) the date of the maximum EVI value, an indicator of the growing season peak, as it indicates the most productive month during the year (phenology indicator) (see^26^ for details).

### Modelling framework

**Figure 2:**
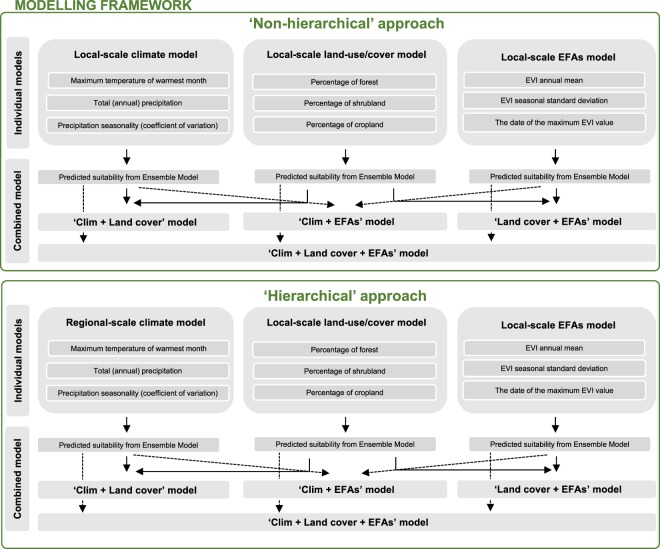
Flow diagram of the modelling approach and steps (see Material and Methods for a detailed description of each step).

We first developed three sets of trivariate models based exclusively upon each type of environmental predictor (henceforth ‘individual’ models): (1) Climate, (2) Land cover, and (3) EFAs (see Fig. 2 for a flow diagram of the modelling approach and steps). We also developed an additional set of models by using the environmental suitability predicted from the ‘individual’ models as predictor variables to integrate in a balanced way all possible combinations of these three environmental drivers within the modelling framework (henceforth ‘combined’ models) (see e.g.^30,48,49^): (4) Climate + Land cover, (5) Climate + EFAs; (6) Land cover + EFAs, and (7) Climate + Land cover + EFAs (Fig. 2). Both individual and combined ENMs were fitted with a maximum number of three variables to control model complexity, increase model transferability^17,61^, and make model performance and transferability comparable^61^ (Fig. 2). Climate models were calibrated at local (Gerês-Xurés) scale at 230 m resolution (henceforth ‘local climate models’) and then combined with the environmental suitability predicted from ENMs based upon land cover and/or EFAs (henceforth ‘non-hierarchical approach’). Climate models were also calibrated at the Iberian Peninsula scale (henceforth ‘regional climate models’) at 10-km resolution. These regional climate models were then directly projected (‘direct downscaling approach’, see^62–64^) to the extent and resolution of Gerês-Xurés Mountains for both 2000 and 2010. These local climate projections were used as predictor variables into combined models to hierarchically integrate climate suitability calibrated at regional scale with the environmental suitability predicted from ENMs based upon land cover and/or EFAs at local scale (henceforth ‘hierarchical approach’, *sensu*^41,42^).

All ENMs were calibrated using seven modelling algorithms available in the R package ‘Biomod2’, version 3.3–7^65^: generalized linear models (GLM), generalized additive models (GAM), generalized boosted regression models (GBM), random forest (RF), factorized distribution algorithm (FDA), multivariate adaptive regression splines (MARS) and artificial neural networks (ANN). For each technique, we used the default settings in biomod2 because these settings are optimized for SDMs (see^66^). Presence-background modelling techniques such as MaxEnt or GARP were not considered as they are designed to cope with the lack of absence data^67^, which is not the case, and did not show significant improvement in terms of predictive accuracy. We applied a 30-fold split-sample procedure by randomly selecting 70% of the data for calibration and the remaining 30% for model evaluation (hereafter ‘crossvalitation’). Our results for the area under the curve (AUC) of the receiver-operating characteristic (ROC), true skill statistic (TSS), the Cohen’s kappa coefficient were highly correlated (see Supplementary Table S3.1), and consequently, we only present results on AUC − a threshold-independent method − as a means of evaluating model performance^68^. In order to quantify model ability to accurately predict presences, we also computed sensitivity (i.e. the proportion of presences that were correctly predicted) as indicator of omission errors^68^. We applied the weighted average approach for computing a consensus (hereafter ‘ensemble model’) of the single models by calculating the weighted average of all single models with AUC > 0.65, using AUC values as model weights. This consensus method provides significantly more robust predictions than all the single-models and other consensus methods^69^. Models with AUC higher than 0.65 (instead of the more usual 0.7)^70^ were considered in the consensus method in order to obtain an ensemble model for all species, thereby ensuring a meaningful set of results to test our hypotheses and avoiding misleading conclusions that might result from considering only AUC values for those species with the highest model accuracies.

### Model transferability

The temporal transferability of the ENMs (i.e. the ability of the model to predict species distribution in a different time frame) was tested by comparing the environmental suitability predicted for 2010 − derived from ensemble models calibrated for 2000 − against observed occurrence data available for 2010 from two independent sources: (1) a dataset obtained from the replication in 2010 of a subset of the initial point counts used to calibrate the models, which enables a temporally (but not spatially) independent assessment of model transferability (i.e. ‘Internal TT’); and (2) another dataset obtained from a distinct set of locations and using a different sampling methodology, which offers a more independent − spatially and temporally − evaluation of the model transferability (i.e. ‘External TT’).

As described above, the AUC of the ROC and Sensitivity were also used as accuracy metrics to evaluate model transferability^68^. We also calculated the number of species with AUC higher than 0.7 as a complementary indicator. To compare the spatial predictions for years 2000 and 2010, derived from the different ENMs, we calculated the Schoener’s D metric, the most common measure of niche overlap^71,72^. In particular, we computed niche overlap between spatial predictions derived from ‘individual’ and ‘combined’ models for each species using the function ‘nicheOverlap’ available in the R package ‘dismo’, version 1.1–4^57^. Then, the Schoener’s D metric values were summarised by computing the average across species for each type of model.

### Hypothesis testing

Each hypothesis and sub-hypothesis was tested by comparing the AUC values (see Table 1 for a detailed description) between the following:

(H1) crossvalidation and temporal transferability assessments (i.e. ‘Internal TT’ and ‘External TT’),

(H2) model types (i.e. ‘individual’ vs ‘combined’; e.g. ‘climate’ vs ‘climate + land cover’),

(H3) modelling approaches (i.e. ‘hierarchical’ vs ‘non-hierarchical’), and

(H4) species traits (e.g. ‘sedentary’ vs ‘migratory’).

We used box plots to display the variability of predictive accuracy obtained from crossvalidation, ‘Internal TT’ and ‘External TT’ assessments, across all predictor combinations (i.e. from ‘individual’ to ‘combined’ models), the two modelling approaches (i.e. ‘hierarchical’ and ‘non-hierarchical’) and all species traits (see Table 2). All plots were constructed with R software and the *ggplot2* package^73^.

In addition, the effect of environmental predictor, modelling approach and species traits on AUC values were estimated using GLMs with a Gaussian error distribution and the ‘identity’ link function^74^ separately for crossvalidation, ‘TT Internal’ and ‘TT External’. In particular, we fitted a set of competing models and applied the multimodel inference approach^75^. We applied a data dredging analysis (the *dredge* function, available in the R package *MuMIn*; R Core Team 2015) to run the GLMs for all (valid) combinations of environmental predictors (interactions between predictor, modelling approach and species trait not included). For each model, we calculated the Akaike Information Criterion (AIC) and ∆_*I*_, where ∆_*I*_ = AIC_i_−AIC_minimum_. All the models with ∆_*I*_ < 7 were considered to have support^75^. The importance of each predictor was obtained by adding the Akaike weights (W_i_) to the models in which that variable is present^76^. The addition of the weights of each variable was considered consequential when ΣW_i_ > 0.5, meaning that half or more of the total Akaike weight for the model set was represented by models that contained that variable^77^. The AUC values for species traits were compared by a Wilcoxon signed rank test for paired samples. To be conservative, we only considered significant changes associated with *p*-values smaller than 0.001.

## Results

### Model transferability hypothesis (H1)

**Figure 3:**
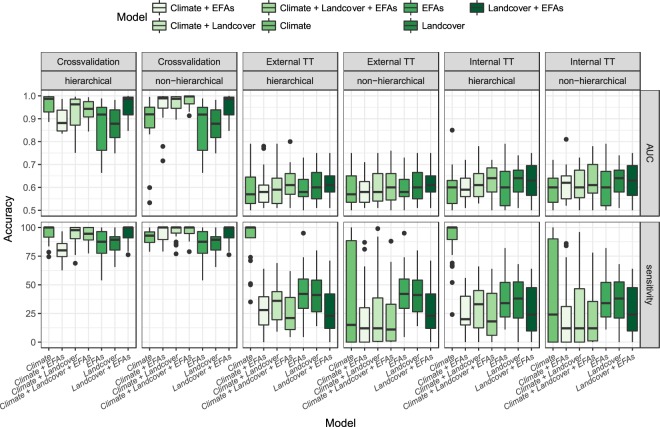
Model performance and transferability measured by AUC and sensitivity values for each set of predictors, modelling approach, and type of evaluation. For all box plots, lower and upper whiskers encompass the 95% interval, lower and upper hinges indicate the first and third quartiles, and the central black line indicates the median value.

All ENMs performed well during the crossvalidation procedure (AUC_mean_ = 0.923 ± 0.081). However, the predictive accuracy of their temporal projections was much lower when tested against temporally (i.e. ‘Internal TT’; AUC_mean_ = 0.615 ± 0.071) and spatiotemporally (i.e. ‘External TT’; AUC_mean_ = 0.601 ± 0.067) independent datasets (Fig. 3). The low temporal transferability (AUC was higher than 0.7 for less than 25% of species) indicates that the ability of the models to predict distributional shifts is limited.

### Environmental-predictor hypothesis (H2)

**Figure 4:**
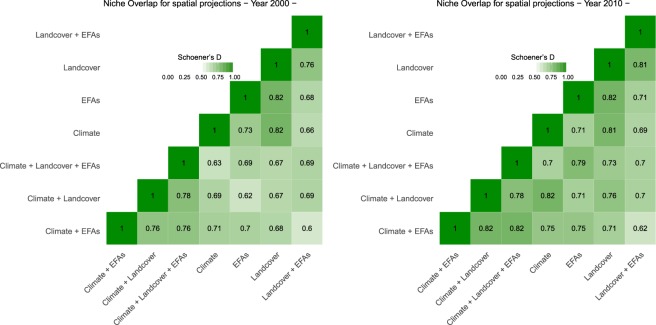
The Schoener’s D values estimated from the spatial predictions derived from the different ENMs for both year 2000 and 2010.

ENMs developed with the three sets of predictors were all useful for describing the distribution of the target species (AUC_Climate_ = 0.889 ± 0.104; AUC_EFA_ = 0.867 ± 0.117; AUC_LCT_ = 0.873 ± 0.071; Fig. 3). The high niche overlap between projections indicates that the different ENMs produce congruent predictions for our target species distributions (Fig. 4). However, spatial dissimilarities were also found, which confirms the complementary spatial information that ENMs based upon the different types of predictors can provide (Schoener’s D values ranging from 0.60 to 0.82, Fig. 4). Models based upon EFAs showed higher niche overlap with combined models (Schoener’s D = 0.69–0.79) than land cover (Schoener’s D = 0.67–0.73) or climate models (Schoener’s D = 0.63–0.70; Fig. 4). The gradual integration of information derived from ENMs based upon the different sets of predictors substantially increased model performance, both within (AUC_mean_ up to 0.98; Fig. 3; ΣW_PRED_ = 0.99; Table 3) but also beyond (Fig. 3; ΣW_PRED_ = 0.98; see ‘Internal TT’ in Table 3; ΣW_PRED_ = 0.99; see ‘TT External’ in Table 3) the calibration time frame. However, despite the enhanced model performance, the overall accuracy of temporal projections remained very low (Fig. 3).

**Table 3:** Model ranking according to ∆AIC (delta Akaike Information Criterion; only models ∆AIC < 7 are shown) for each evaluation type.

Evaluation type	Models	AIC	Delta (∆AIC)	Weight
Crossvalidation	PRED + APP + TRAIT	−3448.3	0	0.908
	PRED + TRAIT	−3443.7	4.598	0.056
Internal TT	PRED	−3684.6	0	0.674
	PRED + APP	−3682.8	1.836	0.268
	PRED + TRAIT	−3679.1	5.516	0.042
External TT	PRED + TRAIT	−3906.3	0	0.718
	PRED + APP + TRAIT	−3904.4	1.877	0.281

Abbreviations: PRED (set of predictors); APP (modelling approach: ‘hierarchical’ vs ‘non-hierarchical’); TRAIT (species trait, see Table 2).

### Hierarchical-integration hypothesis (H3)

The hierarchical integration of climate suitability predicted from regional climate models (calibrated at Iberian level) with the environmental suitability predicted from local ENMs (calibrated at Gerês-Xurés level, based upon land use/cover and ecosystem functioning variables) significantly improved our predictions within (AUC_mean_ up to 0.97; Fig. 3; ΣW_APP_ = 0.90; Table 3), but not beyond the model calibration time period (Fig. 3; ΣW_APP_ = 0.26; see ‘Internal TT’ in Table 3; ΣW_APP_ = 0.28; see ‘External TT’ in Table 3).

### Species-traits hypothesis (H4)

**Figure 5:**
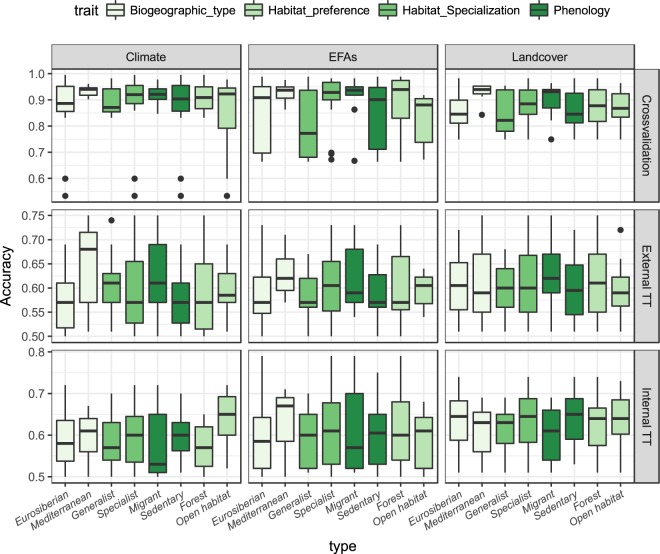
Model performance and transferability measured by AUC values aggregated by species traits for each set of predictors, modelling approach, and type of evaluation. For all box plots, lower and upper whiskers encompass the 95% interval, lower and upper hinges indicate the first and third quartiles, and the central black line indicates the median value.

The species traits significantly affected our ability to accurately describe species distribution within the model calibration time period (Fig. 5; ΣW_TRAIT_ = 0.99; Table 3) and also to predict distributional shifts over time (Fig. 5; ΣW_TRAIT_ = 0.99; see ‘External TT’ in Table 3). However, this result was not consistent when temporal projections were tested against temporally (but not spatially) independent datasets (ΣW_TRAIT_ = 0.04; see ‘Internal TT’ in Table 3).

Model performance and transferability were higher for Mediterranean than for Eurosiberian species (‘H4.1’; crossvalidation: AUC_mean_ = 0.96 vs 0.91, respectively; *p* < 0.001; ‘External TT’ AUC_mean_ = 0.63 vs 0.59, respectively; *p* < 0.001), especially for ENMs developed with climate variables (Fig. 5), except for ‘Internal TT’ evaluations (AUC_mean_ = 0.61 vs 0.61, respectively; *p* = 0.753).

Model performance within crossvalidation was higher for habitat specialist than generalist species (AUC_mean_ = 0.93 vs 0.90, respectively; p < 0.001). However, these species traits did not show any significant effect on temporal transferability (‘H4.2’; ‘Internal TT’ AUC_mean_ = 0.60 vs 0.59, respectively; *p* = 0.043; ‘External TT’ AUC_mean_ = 0.62 vs 0.60, respectively; *p* = 0.223; Fig. 5).

No significant differences were found between forest and open-habitat dwelling species, in either the crossvalidation (AUC_mean_ = 0.93 vs 0.90, respectively; *p* = 0.003) or in the temporal transferability (H4.2; ‘Internal TT’ AUC_mean_ = 0.61 vs 0.62, respectively; *p* = 0.049; ‘TT External’ AUC_mean_ = 0.60 vs 0.59, respectively; *p* = 0.378; Fig. 5). In addition, migrant species were more accurately predicted than sedentary species (AUC_mean_ = 0.94 vs 0.91, respectively; *p* < 0.001), especially with ENMs based upon EFAs (‘H4.3’; Fig. 5), but only within the calibration time period (‘Internal TT’ AUC_mean_ = 0.61 vs 0.60, respectively; *p* = 0.035; ‘External TT’ AUC_mean_ = 0.62 vs 0.59, respectively; *p* = 0.001).

Overall, species traits affecting the performance and transferability of ENMs based upon climate and land cover variables also affected the predictive accuracy of ENMs based upon EFAs (‘H4.4’; see Fig. 5).

## Discussion

The present study compares - for the first time - the performance and temporal transferability of ecological niche models (ENMs) based upon climate, land-use/cover and ecosystem functional attributes (‘EFAs’), as well as the combinations of these factors. It is also, to our knowledge, the first study to evaluate the ability of an approach that hierarchically integrates climate with land-use/cover and ecosystem functional variables (cf.^30,31^) to improve bird distribution predictions beyond the calibration time frame. In addition, this study provides new insights into the relative role of species traits in model performance, being one the few attempts so far to examine the effects on model transferability (but see^38,40,78,79^).

### Integration of predictors and model transferability

Our findings showed that ENMs developed with the three sets of predictors (namely climate, land-use/cover and EFAs) were all useful for describing the distribution of our target species (cf. Figs 3 and S3.1). The integration of these ecologically relevant predictors significantly increased the model performance within the calibration time period, under both the hierarchical and non-hierarchical integration approach (see ‘crossvalidation’ in Fig. 3, Table 3). As expected, the enhanced model performance within the calibration time was also translated into an increase in model transferability (cf. Fig. 3), providing additional support to the relevance of integrating climate and land-use/cover variables to improve our future biodiversity projections^19–21^ (‘H2’). In this sense, the integration of predictor variables related to ecosystem functioning − an often neglected dimension of ecological niche of species (but see^27,80^) − was found to increase model performance and transferability (cf. Fig. 3), especially for migrant, forest specialist species (cf. Fig. 5). Nonetheless, the overall predictive accuracy beyond the calibration time period was considerably low, being acceptable for less than 25% of modelled species (‘H1’). The lack of model transferability might be due to the existence of non-analogous conditions and, consequently, the need to extrapolate^14,81,82^. Then, low transferability is almost a consequence of high uncertainty due to extrapolation^83^. Indeed, a large spatial dissimilarity between years 2000 and 2010 was found for annual precipitation and precipitation seasonality. Theoretically, more temporally stable variables could lead to higher model transferability, but it depends upon the species to be modelled and the relative importance of those variables in the model (see Supplementary Fig. S4.1). Also, land abandonment process and wildfires has been affecting landscape over the last decade (see Supplementary Figs S4.2 and S4.3), with strong impacts on the bird community, as has been already documented for our study area^46,47^. In this sense, the explicit consideration of ecologically meaningful processes for species (i.e., fire disturbance) and more mechanistic approaches when modelling species distributions might have improved the temporal transferability of our ENMs, as has been illustrated for fire-prone systems^84,85^. In addition, the development of more appropriate extrapolation methods can help generate more realistic predictions in different spaces or times, allowing higher model transferability and credibility^86^.

**Figure 6:**
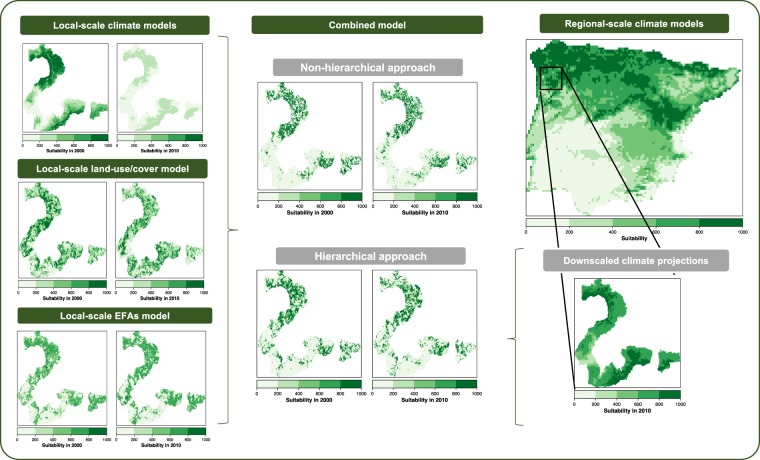
Spatial projections depicting the suitability values predicted for the Eurasian Skylark (Alauda arvensis) from each type of ENM under the hierarchical and non-hierarchical modelling approach for both year 2000 and 2010.

Theoretically, the hierarchical approach should have improved our predictions beyond the calibration time period, as regional climate models (i.e. those calibrated at Iberian scale and projected into the Gerês-Xurés conditions) should be able to capture a wider range of species distributions and, in turn, a wider range of climate conditions than local climate models (i.e. those calibrated at Gerês-Xurés level) (see spatial projections for Eurasian skylark, *Alauda arvensis*, in Fig. 6). Indeed, the high sensitivity of the downscaled climate projections derived from regional models (see Fig. 3) suggests that they were able to capture the broad climate envelope of the species, being refined with the subsequent integration of ENMs based upon land cover and ecosystem functioning variables at Gerês-Xurés scale (cf. Fig. 3). These results contribute to the mounting evidence that hierarchical approaches effectively capture the intrinsic characteristics of ecological systems^41^, successfully integrating climate suitability over large scales with habitat characteristics at more local scales (see e.g.^48,61,87,88^). This multiscale hierarchical approach did not, however, show any advantages in terms of model transferability relative to the classic, non-hierarchical integrative approach (Table 3, Fig. 3), undermining its suitability for making predictions of bird distributions under global change scenarios, at least in our particular case (‘H3’). Here we applied the ‘direct approach’ to obtain fine-grained, downscaled climate suitability from models calibrated at broader scale^62–64^. This approach relies on the assumption ﻿that fine-grain species distributions show the same environmental associations as distributions at the coarse grain^62,63^, which might not be necessarily the case for all species. Alternative hierarchical approaches based on Bayesian modelling framework might help to overcome these limitations^89^, eventually leading to more accurate predictions. Moreover, niche truncation is one of the most challenging issues when ENMs are used to predict in other times or spaces^14,90^. For example, niche truncation can result from a limited range of environmental conditions existing in the study region used to calibrate models (i.e., realized environmental space *sensu*^91^). In our study, models calibrated at local (Gerês-Xurés) and regional (Iberian Peninsula) scales have likely resulted in different niche truncation. Then, the challenge for predictive ecology is how to deal with predictions into non-analogous conditions (i.e., beyond the point of niche truncation), which can be an important constraint, even having chosen the most appropriate predictors. Recent research has also highlighted that the poor performance of uncorrected (random) cross-validation might be caused by dependence structures in the data that might also persist in model residuals, violating the assumption of independence. The large difference found between model accuracy within and beyond the calibration timeframe draws attention to the need for using independent validation data or, alternatively, block cross-validation methods if the goal is predicting to new data or predictor space^92^. Last but not least, the lack of repeated temporal sample structure in our bird data (both at local and regional scale), required for accounting statistically for biases related to imperfect detection, is an additional source of variation^93,94^.

### Species traits and model transferability

Our findings showed that species traits have a significant impact on both model performance and transferability (‘H4’; cf. Fig. 5), despite the large variability among species (see Supplementary Fig. S3). These results confirm the important role of species ecology on model performance, as previously suggested^32,38,40,78,79^. However, our results also suggest that the effects of species traits on predictive accuracy within the model calibration time period are not necessarily translated into its temporal transferability (cf. Table 3; Fig. 5). Thus, while biogeographic origin was found to affect model performance and transferability, habitat specialization and migratory status only impacted model performance (not transferability), and habitat preference did not impact either model performance or transferability (cf. Table 3; Fig. 5). Interestingly, the results also indicate a trait-predictor relationship as some specific traits seem to be linked to specific environmental predictors. For instance, model performance was higher for habitat specialists than for generalist species^95,96^ (see ‘crossvalidation’ in Fig. 5). Generalist species often have local subpopulations that differ in ecological characteristics, so modelling all these subpopulations together could overestimate the species ecological breadth and hence reduce model accuracy^96^. On the contrary, specialist species that inhabit in more restricted environmental conditions are usually more accurately predicted, as long as these conditions are not widely distributed^95^. Similarly, Mediterranean species showed higher predictive accuracy than Eurosiberian species, especially in ENMs that included climate variables (see ‘External TT’ in Fig. 5; ‘H4.1’), due also to a narrower niche and smaller range size in the study area^97^. Hence, our ability to properly model species distributions hinges upon the niche breadth–range size relationship^97^, even though this does not necessarily have to affect model transferability (‘H4.2’; Fig. 5). In fact, some studies found greater model transferability for wide-ranging organisms with broad environmental niches than for narrow-ranging specialists^14,40^. In addition, our results showed that migrant species were more accurately predicted than sedentary species (‘H4.3’; Fig. 5). This is consistent with recent studies that demonstrated that migrating birds track vegetation dynamics^28^; i.e., the birds move with the seasonally progressing green-up of vegetation^98,99^. Food availability is one of the most important resources determining breeding success. On the basis of the well-established principle that resources availability is related to primary productivity^100^, several studies successfully correlated species distributions with the inter-annual variability and seasonal dynamics of vegetation, measured through satellite-derived indices^27,80,101^.

It is particularly interesting to note that species traits affecting the performance and transferability of ENMs based upon climate and land cover variables also affected the predictive accuracy of ENMs based upon EFAs (H4.4, Fig. 5). This may be explained by the fact that EFAs can be directly affected by climate and land use/cover change^25^ and can thus potentially provide information about the integrative response of species to global environmental change^27,102^. Indeed, ENMs based exclusively upon EFAs showed higher niche overlap with the combined models – that integrate the three types of environmental predictors – than land cover or climate models (Fig. 4). These results suggest that satellite-derived EFAs, in addition to assisting the on-going assessment of essential biodiversity variables (EBVs) related to ecosystem functioning^22^ (see also Working Group Ecosystem function GEOBON, http://geobon.org/working-groups/ecosystem-function/), can also contribute substantially to near real-time biodiversity monitoring. Our results therefore confirm the ability of EFAs to accurately model bird distribution – especially for migrant, forest specialist species – although they cannot ensure model transferability by themselves (Fig. 5). Their integration with other ecologically relevant factors, such as climate and land use changes^39,79^, into ENMs will be key to improve temporal predictions^20^.

In conclusion, the study findings confirm the important role of species traits and environmental predictors in model performance and transferability for bird distributions, especially in highly dynamic systems. The progressive integration of ecologically relevant predictors related to climate, land-use/cover and ecosystem functional conditions significantly enhanced model performance within the calibration time period, under both hierarchical and non-hierarchical integration approaches. These findings provide additional support to the importance of integrating climate, land-use/cover and ecosystem functional variables to improve our future biodiversity projections. Unfortunately, the hierarchical multiscale approach did not show substantial advantages in terms of model transferability in comparison with the classic, non-hierarchical integrative approach, undermining its suitability for predicting bird distribution under global change scenarios. We strongly emphasize the importance of considering model transferability, in addition to traditional measures of model accuracy, and the need for caution when using ENMs to predict shifts in bird distributions as high discriminatory power within the calibration time frame does not guarantee the predictive ability of a model. Our findings also showed that species traits may significantly impact both model performance and transferability. In particular, the accuracy of prediction was highest for Mediterranean, migrant, habitat-specialist species, which provides guidance for the suitability of ENMs as an approach for predicting global change responses for birds in our region. However, the findings also suggest that the positive effects of species traits on predictive accuracy within model calibration are not necessarily translated into higher temporal transferability.

## Supplementary information

Supplementary Info S1

Supplementary Info S2

Supplementary Info S3

Supplementary Info S4

Dataset 1

Dataset 2

## Data Availability

The bird datasets and environmental variables used during this study are available in Supplementary Information Files.
