# Supplementary material for: Effects of species traits and environmental predictors on performance and transferability of ecological niche models
Source: Sci Rep. 2019 Mar 12;9:4221. doi: 10.1038/s41598-019-40766-5 (PMC6414724; doi:10.1038/s41598-019-40766-5)
Supplement: Supplementary file 1 — Supplementary Info S1 [file 41598_2019_40766_MOESM1_ESM.docx]

**Online supplementary material**

**Effects of species traits and environmental predictors on performance and transferability of ecological niche models**

Adrián Regos^1,2*^, Laura Gagne^3^, Domingo Alcaraz-Segura^4,5^, João P. Honrado^2,6†^, Jesús Domínguez^1†^

**APPENDIX S1**

This appendix shows the spatial representation, and collinearity structure of the environmental predictors for both 2000 and 2010. The spatial representation of each predictor at 230-m resolution across the study area was done with the R package ‘raster’ (Figs. S1.1 and S2.1). The correlation matrices were computed for the initial set of 19 climate variables (see Table S1.1. and Figs. S1.3 and S1.4), and also for all predictors finally selected for modelling (see Table S1.2. and Figs. S1.5 and S1.6). Correlation matrices are visualized through heatmaps, performed with ‘ggplot2’ package (R Core Team, 2015).

| Name of the climate variables | Acronym |
| --- | --- |
| Mean annual temperature | **Bio1** |
| Mean diurnal range (mean of max temp - min temp | **Bio2** |
| Isothermality (bio2/bio7) (* 100) | **Bio3** |
| Temperature seasonality (standard deviation *100) | **Bio4** |
| Max temperature of warmest month | **Bio5*** |
| Min temperature of coldest month | **Bio6** |
| Temperature annual range (bio5-bio6) | **Bio7** |
| Mean temperature of the wettest quarter | **Bio8** |
| Mean temperature of driest quarter | **Bio9** |
| Mean temperature of warmest quarter | **Bio10** |
| Mean temperature of coldest quarter | **Bio11** |
| Total (annual) precipitation | **Bio12*** |
| Precipitation of wettest month | **Bio13** |
| Precipitation of driest month | **Bio14** |
| Precipitation seasonality (coefficient of variation) | **Bio15*** |
| Precipitation of wettest quarter | **Bio16** |
| Precipitation of driest quarter | **Bio17** |
| Precipitation of warmest quarter | **Bio18** |

**Table S1.1**. Description and acronyms of the 19 climate variables computed for climate models.

| Type | Predictors |
| --- | --- |
| Climate | Maximum temperature of warmest month |
|  | Total (annual) precipitation |
|  | Precipitation seasonality (coefficient of variation) |
| Ecosystem functioning | Productivity indicator: EVI annual mean, an estimator of annual primary production. |
|  | Seasonality indicator: EVI seasonal standard deviation, a descriptor of the difference in carbon gains between season. |
|  | Phenological indicator: the date of the maximum EVI value, an indicator for the peak of the growing season, i.e. indicates the more productive month during the year. |
| Land use/cover | Percentage of forest |
|  | Percentage of shrublands |
|  | Percentage of croplands |

**Table S1.2**. Description and acronyms of the 9 environmental predictors finally selected for modelling.

**Fig. S1.1** Spatial representation of each type of predictors for year 2000. See table S1.2 for description.

**Fig. S1.2** Spatial representation of each type of predictors for year 2010. See table S1.2 for description.

**Fig. S1.3** Correlation matrix heatmaps for the 19 climate variables for year 2000.

**Fig. S1.4** Correlation matrix heatmaps for the 19 climate variables for year 2010.

**Fig. S1.5** Correlation matrix heatmaps for the 9 environmental predictors for year 2000. See table S1.2 for description.

**Fig. S1.5** Correlation matrix heatmaps for the 9 environmental predictors for year 2010. See table S1.2 for description.
