# Supplementary material for: Effects of species traits and environmental predictors on performance and transferability of ecological niche models
Source: Sci Rep. 2019 Mar 12;9:4221. doi: 10.1038/s41598-019-40766-5 (PMC6414724; doi:10.1038/s41598-019-40766-5)
Supplement: Supplementary file 2 — Supplementary Info S2 [file 41598_2019_40766_MOESM2_ESM.docx]

**Online supplementary material**

**Effects of species traits and environmental predictors on performance and transferability of ecological niche models**

Adrián Regos^1,2*^, Laura Gagne^3^, Domingo Alcaraz-Segura^4,5^, João P. Honrado^2,6†^, Jesús Domínguez^1†^

**APPENDIX S2**

In this appendix, we describe the technical details regarding the preprocessing the Landsat images, definition of training/test areas, classification procedures and evaluation tests of the land use/cover maps. The land use and cover maps were derived from a range of cloud-free Landsat images (30-m resolution) obtained for years 2000 and 2010. Specifically, the solar and thermal multispectral bands of Landsat TM and ETM+ images were acquired for the same temporal sequence as the bird sampling was carried out (June 8th 2000, June 24th 2000, March 20th 2000, May 19th 2010 and July 30th 2010). The Landsat data were downloaded from the United States Geological Survey ([http://glovis.usgs.gov](#_Hlk309846099)) with a processing level (LT1) that included a geometric correction performed with ground control points and digital terrain model (RMS average of 4.04 m). The data were projected in the Universal Transverse Mercator (UTM) coordinate system (World Geodetic System 84 datum, projection UTM Zone 29 North). The images were also radiometrically calibrated according to the method described by Pons and Solé-Sugrañes (1994), which uses a Digital Elevation Model ([http://www.gdem.aster.ersdac.or.jp](#_Hlk310352638)) to avoid cast- and self-shadows and different illumination effects caused by the intense topographic variations in the study area.

The land cover classification was obtained using a hybrid classifier, a combination of unsupervised and supervised strategies [for methodological details, see Serra et al. (2003); Serra et al. (2008)]. The procedure involves both unsupervised classification and training areas. In the unsupervised classification, the spectral classes of pixels are first identified by cluster analysis. This step included a non-hierarchical clustering algorithm commonly used in remote sensing (Interactive Self Organizing Data Analysis, ISODATA). The spectral classes obtained by the ISODATA algorithm were then assigned to LULC categories by considering the training areas. Identification of training areas for each category was supported by different RGB (Red, Green and Blue) composites (obtained by combining satellite bands), airborne photography and fieldwork. Digital orthophotography (natural colour, scale 1:18,000 scale) were acquired from the *Plan Nacional de Ortofotografía Aérea* (<http://www.ign.es/>). Prior knowledge of the Xurés Mountains acquired during the fieldwork enhanced the process of defining training areas, thus improving the overall accuracy of the land cover maps.

The accuracy of the maps was assessed by using confusion matrices and application of the overall accuracy and the kappa coefficient (Foody 2002) (Tab1e S2.1). These land-use/cover maps were then reclassified into three broad categories: (1) scrubland, (2) forest and (3) cropland. We calculated the percentage of each land cover type in each geographic unit (i.e. grid cell). Land-cover variables were resampled to the original resolution of EFAs (~ 230 meters).

**Table S2.1.** Confusion matrix and statistic accuracy assessment for the classification. Both classification results (in row) and ground truth (in column) are expressed in pixels. **OE**: omission errors (%), **PrA**: producer's accuracy (%), **CoE**: commission errors (%), **UA**: user's accuracy (%). **BrG**: Bare Ground, **OSh**: Open Shrubland, **Wat**: Water, **EvF**: Evergreen Forest, **CSh**: Closed Shrubland, **DeF**: Deciduous Forest, **Crp**: Cropland, **Urb**: Urban settlements.

| **2000** | **BrG** | **OSh** | **Wat** | **EvF** | **CSh** | **DeF** | **Crp** | **Urb** | **Total** | **CoE** | **UA** |
| --- | --- | --- | --- | --- | --- | --- | --- | --- | --- | --- | --- |
| **BrG** | 493 | 52 | 0 | 0 | 0 | 0 | 2 | 0 | 547 | 9.87 | 90.13 |
| **OSh** | 41 | 519 | 0 | 1 | 115 | 0 | 1 | 2 | 679 | 23.56 | 76.44 |
| **Wat** | 0 | 0 | 1463 | 0 | 0 | 0 | 0 | 0 | 1463 | 0 | 100 |
| **EvF** | 0 | 0 | 0 | 634 | 45 | 0 | 0 | 1 | 680 | 6.76 | 93.24 |
| **CSh** | 0 | 54 | 0 | 9 | 840 | 82 | 30 | 4 | 1019 | 17.57 | 82.43 |
| **DeF** | 0 | 0 | 0 | 0 | 109 | 421 | 7 | 0 | 537 | 21.6 | 78.4 |
| **Crp** | 0 | 2 | 0 | 0 | 0 | 9 | 150 | 0 | 161 | 6.83 | 93.17 |
| **Urb** | 0 | 0 | 0 | 0 | 0 | 0 | 0 | 1442 | 1442 | 0 | 100 |
| **Total** | 534 | 627 | 1463 | 644 | 1109 | 512 | 190 | 1449 | 6528 | Overall accuracy=91.33% | |
| **OE** | 7.68 | 17.22 | 0 | 1.55 | 24.26 | 17.77 | 21.05 | 0.48 |  | Kappa Index= 0.90 | |
| **PrU** | 92.32 | 82.78 | 100 | 98.45 | 75.74 | 82.23 | 78.95 | 99.52 |  |  |  |
| **2010** | **BrG** | **OSh** | **Wat** | **EvF** | **CSh** | **DeF** | **Crp** | **Urb** | **Total** | **CoE** | **UA** |
| **BrG** | 85 | 11 | 0 | 0 | 0 | 0 | 0 | 0 | 96 | 11.46 | 88.54 |
| **OSh** | 55 | 822 | 0 | 0 | 46 | 49 | 0 | 0 | 1284 | 0 | 100 |
| **Wat** | 0 | 0 | 1284 | 0 | 0 | 0 | 0 | 0 | 972 | 15.43 | 84.57 |
| **EvF** | 0 | 0 | 0 | 674 | 1 | 1 | 0 | 0 | 676 | 0.3 | 99.7 |
| **CSh** | 0 | 4 | 0 | 4 | 236 | 95 | 5 | 0 | 239 | 34.73 | 65.27 |
| **DeF** | 0 | 0 | 0 | 1 | 0 | 1105 | 38 | 0 | 1144 | 3.41 | 96.59 |
| **Crp** | 0 | 1 | 0 | 0 | 0 | 82 | 156 | 0 | 344 | 31.4 | 68.6 |
| **Urb** | 0 | 0 | 0 | 0 | 0 | 7 | 0 | 449 | 456 | 1.54 | 98.46 |
| **Total** | 140 | 838 | 1284 | 679 | 283 | 1339 | 199 | 449 | 5211 | Overall accuracy=92.32% | |
| **OE** | 39.29 | 1.91 | 0 | 0.74 | 16.61 | 17.48 | 21.61 | 0 |  | Kappa Index= 0.91 | |
| **PrU** | 60.71 | 98.09 | 100 | 99.26 | 83.39 | 82.52 | 78.39 | 100 |  |  | |

**References**

Campbell J. (2008) Introduction to remote sensing, 4th ed. Taylor and Francis, London

Foody GM (2002) Status of land cover classification accuracy assessment. Remote Sens Environ 80:185–201. doi: 10.1016/S0034-4257(01)00295-4

Pons X, Solé-Sugrañes L (1994) A Simple Radiometric Correction Model to Improve Automatic Mapping of Vegetation from Multispectral Satellite Data. Remote Sens Environ 48:191–204.

Serra P, Pons X, Saurí D (2008) Land-cover and land-use change in a Mediterranean landscape: A spatial analysis of driving forces integrating biophysical and human factors. Appl Geogr 28:189–209. doi: 10.1016/j.apgeog.2008.02.001

Serra P, Pons X, Saurı́ D (2003) Post-classification change detection with data from different sensors: some accuracy considerations. Int J Remote Sens 24:3311–3340.
