# Supplementary material for: Effects of species traits and environmental predictors on performance and transferability of ecological niche models
Source: Sci Rep. 2019 Mar 12;9:4221. doi: 10.1038/s41598-019-40766-5 (PMC6414724; doi:10.1038/s41598-019-40766-5)
Supplement: Supplementary file 3 — Supplementary Info S3 [file 41598_2019_40766_MOESM3_ESM.docx]

**Online supplementary material**

**Effects of species traits and environmental predictors on performance and transferability of ecological niche models**

Adrián Regos^1,2*^, Laura Gagne^3^, Domingo Alcaraz-Segura^4,5^, João P. Honrado^2,6†^, Jesús Domínguez^1†^

**APPENDIX S3**

This appendix shows the ENMs performance derived from the individual and combined models measured with five accuracy metrics: (1) the area under the curve (AUC)^1^, ﻿sensitivity (i.e. the percentage of presence correctly predicted), specificity (i.e. the percentage of absence correctly predicted), the true skill statistic (TSS)^2^ and the Cohen’s kappa coefficient^3^ (Table S3.1). The accuracy metrics from the different metrics across species and models were compared using the Pearson’s correlation coefficient. AUC was found to be significantly correlated (p < 0.001) with the rest of the accuracy metrics considered: Pearson_AUC_-_TSS_ = 0.96; Pearson_AUC_-_Kappa_ = 0.89; Pearson_AUC_-_Sensitivity_ = 0.77; and Pearson_AUC_-_Specificity_ = 0.88.

| Species | model | AUC | Sensitivity | Specificity | TSS | Kappa |
| --- | --- | --- | --- | --- | --- | --- |
| CPAL | Climate | 0.95 | 100 | 85.093 | 0.851 | 0.535 |
| STUR | Climate | 0.902 | 100 | 71.429 | 0.711 | 0.377 |
| CCAN | Climate | 0.847 | 81.955 | 76.415 | 0.582 | 0.559 |
| PVIR | Climate | 0.982 | 95.833 | 95.639 | 0.915 | 0.731 |
| AARV | Climate | 0.919 | 93.056 | 79.853 | 0.714 | 0.623 |
| TTRO | Climate | 0.871 | 81.863 | 75.177 | 0.567 | 0.571 |
| PMOD | Climate | 0.856 | 80 | 72.941 | 0.529 | 0.478 |
| ERUB | Climate | 0.854 | 85.714 | 74.468 | 0.591 | 0.455 |
| STOR | Climate | 0.599 | 92.063 | 24.823 | 0.169 | 0.107 |
| TMER | Climate | 0.832 | 78.889 | 74.51 | 0.53 | 0.469 |
| SUND | Climate | 0.94 | 88.095 | 87.129 | 0.747 | 0.592 |
| SCOM | Climate | 0.926 | 95.238 | 76.238 | 0.708 | 0.622 |
| SATR | Climate | 0.862 | 88.095 | 67.816 | 0.559 | 0.555 |
| RIGN | Climate | 0.957 | 96.154 | 87.031 | 0.828 | 0.723 |
| PCRI | Climate | 0.972 | 92.857 | 95.268 | 0.872 | 0.783 |
| PATE | Climate | 0.859 | 90 | 65.283 | 0.544 | 0.517 |
| PMAJ | Climate | 0.885 | 84.211 | 80.982 | 0.652 | 0.407 |
| CBRA | Climate | 0.888 | 100 | 65.231 | 0.646 | 0.344 |
| OORI | Climate | 0.921 | 93.103 | 79.747 | 0.725 | 0.488 |
| LCOL | Climate | 0.978 | 100 | 90.214 | 0.896 | 0.669 |
| GGLA | Climate | 0.995 | 100 | 95.611 | 0.956 | 0.89 |
| FCOE | Climate | 0.922 | 91.765 | 79.615 | 0.706 | 0.658 |
| SSER | Climate | 0.942 | 94.872 | 80.392 | 0.743 | 0.589 |
| CCHL | Climate | 0.909 | 96.154 | 77.743 | 0.739 | 0.444 |
| CCANN | Climate | 0.961 | 96.667 | 88.254 | 0.846 | 0.642 |
| ECIA | Climate | 0.533 | 81.25 | 29.537 | 0.108 | 0.068 |
| PIBE | Climate | 0.95 | 89.583 | 87.205 | 0.768 | 0.68 |
| CPAL | Climate + Landcover | 0.997 | 100 | 97.826 | 0.978 | 0.89 |
| STUR | Climate + Landcover | 0.989 | 100 | 95.031 | 0.95 | 0.767 |
| CCAN | Climate + Landcover | 0.951 | 92.481 | 84.906 | 0.774 | 0.751 |
| PVIR | Climate + Landcover | 0.998 | 100 | 99.065 | 0.991 | 0.936 |
| AARV | Climate + Landcover | 0.982 | 98.611 | 88.645 | 0.873 | 0.823 |
| TTRO | Climate + Landcover | 0.938 | 76.961 | 93.617 | 0.699 | 0.694 |
| PMOD | Climate + Landcover | 0.927 | 92.222 | 77.255 | 0.691 | 0.658 |
| ERUB | Climate + Landcover | 0.936 | 87.302 | 86.17 | 0.735 | 0.666 |
| STOR | Climate + Landcover | 0.896 | 96.825 | 67.376 | 0.642 | 0.567 |
| TMER | Climate + Landcover | 0.942 | 85.556 | 86.667 | 0.718 | 0.721 |
| SUND | Climate + Landcover | 0.978 | 100 | 87.789 | 0.875 | 0.766 |
| SCOM | Climate + Landcover | 0.988 | 100 | 92.739 | 0.927 | 0.803 |
| SATR | Climate + Landcover | 0.938 | 84.524 | 87.356 | 0.719 | 0.688 |
| RIGN | Climate + Landcover | 0.996 | 100 | 95.563 | 0.956 | 0.921 |
| PCRI | Climate + Landcover | 0.997 | 100 | 98.738 | 0.987 | 0.927 |
| PATE | Climate + Landcover | 0.949 | 93.75 | 84.528 | 0.783 | 0.7 |
| PMAJ | Climate + Landcover | 0.971 | 100 | 89.264 | 0.893 | 0.648 |
| CBRA | Climate + Landcover | 0.998 | 100 | 98.462 | 0.982 | 0.889 |
| OORI | Climate + Landcover | 0.991 | 100 | 94.937 | 0.949 | 0.799 |
| LCOL | Climate + Landcover | 0.999 | 100 | 99.388 | 0.994 | 0.944 |
| GGLA | Climate + Landcover | 0.999 | 100 | 99.373 | 0.994 | 0.96 |
| FCOE | Climate + Landcover | 0.971 | 96.471 | 87.308 | 0.838 | 0.783 |
| SSER | Climate + Landcover | 0.994 | 100 | 94.444 | 0.944 | 0.868 |
| CCHL | Climate + Landcover | 0.987 | 100 | 94.357 | 0.94 | 0.776 |
| CCANN | Climate + Landcover | 0.996 | 100 | 97.46 | 0.975 | 0.91 |
| ECIA | Climate + Landcover | 0.919 | 98.438 | 69.751 | 0.678 | 0.645 |
| PIBE | Climate + Landcover | 0.988 | 100 | 92.929 | 0.929 | 0.819 |
| CPAL | Climate + EFAs | 0.999 | 100 | 99.688 | 0.997 | 0.977 |
| STUR | Climate + EFAs | 0.992 | 100 | 97.188 | 0.972 | 0.852 |
| CCAN | Climate + EFAs | 0.905 | 85.606 | 78.673 | 0.643 | 0.629 |
| PVIR | Climate + EFAs | 0.999 | 100 | 99.373 | 0.994 | 0.957 |
| AARV | Climate + EFAs | 0.98 | 95.714 | 89.377 | 0.85 | 0.822 |
| TTRO | Climate + EFAs | 0.939 | 82.843 | 90.647 | 0.735 | 0.717 |
| PMOD | Climate + EFAs | 0.913 | 88.889 | 79.447 | 0.679 | 0.615 |
| ERUB | Climate + EFAs | 0.921 | 90.476 | 78.929 | 0.694 | 0.621 |
| STOR | Climate + EFAs | 0.779 | 79.032 | 62.633 | 0.401 | 0.354 |
| TMER | Climate + EFAs | 0.954 | 90 | 87.747 | 0.777 | 0.741 |
| SUND | Climate + EFAs | 0.985 | 100 | 91.694 | 0.914 | 0.824 |
| SCOM | Climate + EFAs | 0.971 | 97.619 | 88.04 | 0.85 | 0.739 |
| SATR | Climate + EFAs | 0.971 | 86.905 | 94.208 | 0.811 | 0.798 |
| RIGN | Climate + EFAs | 0.998 | 100 | 96.22 | 0.962 | 0.944 |
| PCRI | Climate + EFAs | 0.996 | 100 | 98.095 | 0.981 | 0.904 |
| PATE | Climate + EFAs | 0.917 | 92.5 | 75.665 | 0.678 | 0.622 |
| PMAJ | Climate + EFAs | 0.993 | 100 | 96.605 | 0.966 | 0.848 |
| CBRA | Climate + EFAs | 0.994 | 100 | 96.594 | 0.966 | 0.826 |
| OORI | Climate + EFAs | 0.996 | 100 | 97.771 | 0.978 | 0.881 |
| LCOL | Climate + EFAs | 0.999 | 100 | 99.692 | 0.997 | 0.971 |
| GGLA | Climate + EFAs | 0.999 | 100 | 99.369 | 0.994 | 0.96 |
| FCOE | Climate + EFAs | 0.953 | 88.235 | 89.147 | 0.774 | 0.725 |
| SSER | Climate + EFAs | 0.991 | 100 | 94.408 | 0.941 | 0.855 |
| CCHL | Climate + EFAs | 0.989 | 100 | 94.322 | 0.943 | 0.795 |
| CCANN | Climate + EFAs | 0.99 | 100 | 95.847 | 0.958 | 0.828 |
| ECIA | Climate + EFAs | 0.716 | 82.812 | 50.179 | 0.33 | 0.246 |
| PIBE | Climate + EFAs | 0.995 | 100 | 94.915 | 0.946 | 0.879 |
| CPAL | Climate + Landcover + EFAs | 1 | 100 | 99.688 | 0.997 | 0.977 |
| STUR | Climate + Landcover + EFAs | 0.997 | 100 | 99.062 | 0.991 | 0.934 |
| CCAN | Climate + Landcover + EFAs | 0.956 | 91.667 | 85.782 | 0.77 | 0.749 |
| PVIR | Climate + Landcover + EFAs | 1 | 100 | 100 | 1 | 1 |
| AARV | Climate + Landcover + EFAs | 0.992 | 100 | 91.575 | 0.916 | 0.883 |
| TTRO | Climate + Landcover + EFAs | 0.951 | 78.922 | 95.683 | 0.737 | 0.717 |
| PMOD | Climate + Landcover + EFAs | 0.936 | 90 | 81.423 | 0.711 | 0.658 |
| ERUB | Climate + Landcover + EFAs | 0.947 | 98.413 | 77.5 | 0.759 | 0.692 |
| STOR | Climate + Landcover + EFAs | 0.913 | 98.387 | 70.107 | 0.685 | 0.572 |
| TMER | Climate + Landcover + EFAs | 0.974 | 87.778 | 93.676 | 0.814 | 0.817 |
| SUND | Climate + Landcover + EFAs | 0.99 | 100 | 92.691 | 0.927 | 0.882 |
| SCOM | Climate + Landcover + EFAs | 0.991 | 100 | 94.02 | 0.94 | 0.85 |
| SATR | Climate + Landcover + EFAs | 0.979 | 89.286 | 96.139 | 0.854 | 0.851 |
| RIGN | Climate + Landcover + EFAs | 1 | 98.077 | 99.656 | 0.977 | 0.977 |
| PCRI | Climate + Landcover + EFAs | 0.998 | 100 | 99.048 | 0.99 | 0.944 |
| PATE | Climate + Landcover + EFAs | 0.954 | 92.5 | 87.072 | 0.796 | 0.726 |
| PMAJ | Climate + Landcover + EFAs | 0.998 | 100 | 97.84 | 0.978 | 0.914 |
| CBRA | Climate + Landcover + EFAs | 1 | 100 | 99.69 | 0.997 | 0.974 |
| OORI | Climate + Landcover + EFAs | 0.999 | 100 | 99.045 | 0.99 | 0.946 |
| LCOL | Climate + Landcover + EFAs | 1 | 100 | 100 | 1 | 1 |
| GGLA | Climate + Landcover + EFAs | 1 | 100 | 99.685 | 0.997 | 0.98 |
| FCOE | Climate + Landcover + EFAs | 0.974 | 94.118 | 91.86 | 0.86 | 0.811 |
| SSER | Climate + Landcover + EFAs | 0.998 | 100 | 96.382 | 0.964 | 0.925 |
| CCHL | Climate + Landcover + EFAs | 0.998 | 100 | 97.161 | 0.972 | 0.939 |
| CCANN | Climate + Landcover + EFAs | 0.998 | 100 | 98.403 | 0.984 | 0.929 |
| ECIA | Climate + Landcover + EFAs | 0.93 | 95.312 | 76.703 | 0.72 | 0.631 |
| PIBE | Climate + Landcover + EFAs | 0.998 | 100 | 97.288 | 0.973 | 0.939 |
| CPAL | Landcover | 0.954 | 91.304 | 89.13 | 0.801 | 0.593 |
| STUR | Landcover | 0.939 | 91.304 | 88.509 | 0.795 | 0.542 |
| CCAN | Landcover | 0.822 | 81.955 | 67.925 | 0.499 | 0.482 |
| PVIR | Landcover | 0.938 | 87.5 | 90.031 | 0.773 | 0.531 |
| AARV | Landcover | 0.888 | 95.833 | 68.864 | 0.647 | 0.512 |
| TTRO | Landcover | 0.78 | 69.608 | 73.759 | 0.431 | 0.424 |
| PMOD | Landcover | 0.749 | 65.556 | 72.157 | 0.373 | 0.375 |
| ERUB | Landcover | 0.76 | 73.016 | 65.603 | 0.365 | 0.326 |
| STOR | Landcover | 0.808 | 79.365 | 71.277 | 0.497 | 0.353 |
| TMER | Landcover | 0.813 | 86.667 | 62.745 | 0.493 | 0.487 |
| SUND | Landcover | 0.843 | 73.81 | 80.528 | 0.543 | 0.408 |
| SCOM | Landcover | 0.913 | 83.333 | 85.149 | 0.676 | 0.608 |
| SATR | Landcover | 0.749 | 73.81 | 69.349 | 0.432 | 0.35 |
| RIGN | Landcover | 0.882 | 90.385 | 76.792 | 0.672 | 0.495 |
| PCRI | Landcover | 0.878 | 89.286 | 72.555 | 0.615 | 0.479 |
| PATE | Landcover | 0.844 | 81.25 | 73.208 | 0.541 | 0.465 |
| PMAJ | Landcover | 0.812 | 78.947 | 71.166 | 0.501 | 0.292 |
| CBRA | Landcover | 0.982 | 95 | 96.308 | 0.907 | 0.727 |
| OORI | Landcover | 0.931 | 86.207 | 92.405 | 0.786 | 0.599 |
| LCOL | Landcover | 0.964 | 100 | 91.743 | 0.917 | 0.537 |
| GGLA | Landcover | 0.939 | 92.308 | 85.58 | 0.779 | 0.5 |
| FCOE | Landcover | 0.84 | 90.588 | 62.692 | 0.528 | 0.474 |
| SSER | Landcover | 0.951 | 97.436 | 84.967 | 0.805 | 0.625 |
| CCHL | Landcover | 0.933 | 100 | 76.176 | 0.759 | 0.478 |
| CCANN | Landcover | 0.955 | 96.667 | 83.175 | 0.798 | 0.619 |
| ECIA | Landcover | 0.847 | 90.625 | 71.53 | 0.606 | 0.449 |
| PIBE | Landcover | 0.869 | 91.667 | 71.044 | 0.614 | 0.442 |
| CPAL | Landcover + EFAs | 0.998 | 100 | 98.438 | 0.984 | 0.931 |
| STUR | Landcover + EFAs | 0.995 | 100 | 98.438 | 0.984 | 0.894 |
| CCAN | Landcover + EFAs | 0.893 | 82.576 | 79.147 | 0.617 | 0.599 |
| PVIR | Landcover + EFAs | 1 | 100 | 100 | 1 | 1 |
| AARV | Landcover + EFAs | 0.974 | 90 | 95.238 | 0.845 | 0.816 |
| TTRO | Landcover + EFAs | 0.883 | 78.922 | 82.734 | 0.602 | 0.595 |
| PMOD | Landcover + EFAs | 0.888 | 81.111 | 79.051 | 0.598 | 0.584 |
| ERUB | Landcover + EFAs | 0.847 | 76.19 | 77.857 | 0.54 | 0.452 |
| STOR | Landcover + EFAs | 0.896 | 95.161 | 70.463 | 0.656 | 0.527 |
| TMER | Landcover + EFAs | 0.959 | 87.778 | 92.885 | 0.803 | 0.78 |
| SUND | Landcover + EFAs | 0.973 | 95.238 | 90.033 | 0.853 | 0.72 |
| SCOM | Landcover + EFAs | 0.974 | 100 | 87.375 | 0.87 | 0.767 |
| SATR | Landcover + EFAs | 0.963 | 97.619 | 83.012 | 0.806 | 0.753 |
| RIGN | Landcover + EFAs | 0.995 | 100 | 94.502 | 0.945 | 0.909 |
| PCRI | Landcover + EFAs | 0.986 | 100 | 94.603 | 0.943 | 0.79 |
| PATE | Landcover + EFAs | 0.91 | 88.75 | 77.186 | 0.656 | 0.6 |
| PMAJ | Landcover + EFAs | 0.991 | 100 | 95.37 | 0.954 | 0.777 |
| CBRA | Landcover + EFAs | 1 | 100 | 99.69 | 0.997 | 0.974 |
| OORI | Landcover + EFAs | 0.995 | 100 | 96.815 | 0.968 | 0.887 |
| LCOL | Landcover + EFAs | 0.999 | 100 | 99.385 | 0.994 | 0.944 |
| GGLA | Landcover + EFAs | 0.994 | 100 | 96.845 | 0.968 | 0.857 |
| FCOE | Landcover + EFAs | 0.913 | 91.765 | 78.682 | 0.704 | 0.611 |
| SSER | Landcover + EFAs | 0.989 | 97.436 | 96.382 | 0.938 | 0.859 |
| CCHL | Landcover + EFAs | 0.992 | 100 | 94.322 | 0.943 | 0.833 |
| CCANN | Landcover + EFAs | 0.992 | 100 | 95.208 | 0.952 | 0.833 |
| ECIA | Landcover + EFAs | 0.921 | 93.75 | 75.986 | 0.697 | 0.606 |
| PIBE | Landcover + EFAs | 0.992 | 100 | 93.22 | 0.929 | 0.886 |
| CPAL | EFAs | 0.949 | 86.957 | 91.562 | 0.785 | 0.715 |
| STUR | EFAs | 0.976 | 100 | 91.562 | 0.916 | 0.72 |
| CCAN | EFAs | 0.667 | 71.212 | 58.294 | 0.295 | 0.276 |
| PVIR | EFAs | 0.988 | 100 | 95.925 | 0.959 | 0.822 |
| AARV | EFAs | 0.899 | 77.143 | 86.447 | 0.625 | 0.584 |
| TTRO | EFAs | 0.772 | 64.706 | 79.137 | 0.438 | 0.418 |
| PMOD | EFAs | 0.752 | 64.444 | 76.68 | 0.405 | 0.377 |
| ERUB | EFAs | 0.664 | 53.968 | 75.357 | 0.293 | 0.235 |
| STOR | EFAs | 0.694 | 88.71 | 41.993 | 0.307 | 0.233 |
| TMER | EFAs | 0.888 | 86.667 | 82.213 | 0.689 | 0.616 |
| SUND | EFAs | 0.91 | 88.095 | 79.402 | 0.672 | 0.557 |
| SCOM | EFAs | 0.863 | 80.952 | 79.402 | 0.6 | 0.393 |
| SATR | EFAs | 0.925 | 91.667 | 78.764 | 0.689 | 0.632 |
| RIGN | EFAs | 0.976 | 90.385 | 96.22 | 0.866 | 0.827 |
| PCRI | EFAs | 0.932 | 89.286 | 85.079 | 0.74 | 0.522 |
| PATE | EFAs | 0.698 | 63.75 | 71.103 | 0.349 | 0.289 |
| PMAJ | EFAs | 0.973 | 100 | 87.346 | 0.867 | 0.663 |
| CBRA | EFAs | 0.978 | 100 | 94.118 | 0.941 | 0.651 |
| OORI | EFAs | 0.982 | 100 | 92.038 | 0.914 | 0.728 |
| LCOL | EFAs | 0.918 | 77.778 | 92.615 | 0.704 | 0.533 |
| GGLA | EFAs | 0.943 | 96.154 | 81.073 | 0.769 | 0.566 |
| FCOE | EFAs | 0.681 | 56.471 | 72.481 | 0.282 | 0.247 |
| SSER | EFAs | 0.937 | 82.051 | 92.105 | 0.735 | 0.667 |
| CCHL | EFAs | 0.95 | 100 | 78.233 | 0.779 | 0.596 |
| CCANN | EFAs | 0.903 | 96.667 | 71.885 | 0.679 | 0.453 |
| ECIA | EFAs | 0.672 | 79.688 | 50.896 | 0.294 | 0.206 |
| PIBE | EFAs | 0.939 | 87.5 | 90.847 | 0.777 | 0.692 |

**Table S3.1.** Accuracy metrics obtained from each type of model across species: (1) the area under the curve (AUC)^1^, sensitivity (i.e. the percentage of presence correctly predicted), specificity (i.e. the percentage of absence correctly predicted), the true skill statistic (TSS)^2^ and the Cohen’s kappa coefficient^3^. See acronyms of each species in Table 2.

This appendix also shows the AUC values for each bird species under the hierarchical and non-hierarchical approach. and each set of predictors (Fig. S3.1). AUC values were obtained by comparing the predicted environmental suitability against the observed occurrence data available obtained from the 5-min point counts for year 2000 (i.e., crossvalidation) (red dots), 5-min point counts for year 2010 (i.e., ‘TT Internal’ dataset) (blue dots), and 20-min point counts for year 2010 (i.e., ‘TT External’ dataset) (green dots).

**Fig. S3.1**. AUC values for each bird species under the hierarchical and non-hierarchical approach, and each set of predictors. See acronyms of each species in Table 2.
