# Supplementary material for: Effects of species traits and environmental predictors on performance and transferability of ecological niche models
Source: Sci Rep. 2019 Mar 12;9:4221. doi: 10.1038/s41598-019-40766-5 (PMC6414724; doi:10.1038/s41598-019-40766-5)
Supplement: Supplementary file 4 — Supplementary Info S4 [file 41598_2019_40766_MOESM4_ESM.docx]

**Online supplementary material**

**Effects of species traits and environmental predictors on performance and transferability of ecological niche models**

Adrián Regos^1,2*^, Laura Gagne^3^, Domingo Alcaraz-Segura^4,5^, João P. Honrado^2,6†^, Jesús Domínguez^1†^

**APPENDIX S4**

This appendix aims to generate a series of maps that depicts the change over time (2000 to 2010) of the landscape, EFAs and climate variables. In particular, we quantified spatially the degree of temporal similarity between 2000 and 2010 for each predictor variable by computing a multivariate environmental similarity surfaces (MESS) analysis using the function ‘mess’ available in the R package ‘dismo’ (Fig. S4.1). We also analysed the land-use/cover changes and area burned by wildfires between 2000 and 2010 to illustrate the impact of these drivers on landscape and EFAs variables and thus infer their potential role on the model transferability. The contribution of each land cover type to net change (i.e., conversion from one land cover type to another) was estimated through a transition matrix obtained by cross-tabulation of the land-use/cover maps. Transition matrices were computed with the R package lulcc v.1.0.2 ^1^ and visualized through a circular plot performed with the circlize R package ^2,3^ (Fig. S4.2). The area burned between 2001 and 2010 was obtained from the Burned Area product derived from the ﻿Moderate Resolution Imaging Spectroradiometer (MODIS) instruments, on-board NASA's Terra and Aqua satellites (Fig. S4.3).

Results from the MESS analysis clearly indicates that the predictor variables with the largest spatial dissimilarities were the climate variables, mainly annual precipitation and precipitation seasonality (Fig. S4.1). However, the inclusion of more temporarily stable climate variables (Fig. S4.2) did not significantly increase temporal transferability (Table S4.1; W = 384.5, p-value = 0.7355). EFAs and land cover variables showed overall positive MESS values, suggesting similarities between both years. However, large areas also showed values close to zero, pointing to areas highly dynamics. Land abandonment (i.e., land cover conversion from cropland to shrubland and from shrubland to forest) and deforestation processes (i.e. conversion from forest to shrubland; Fig. S4.3) likely caused by wildfires (which has affected 25% of the study area between 2000 and 2010; Fig. S4.4) are the main drives affecting landscape and ecosystem functioning changes^4,5^, with potential impacts on model transferability.

**Fig. S4.1.** MESS analysis between 2000 and 2010 for each predictor variable. In the MESS maps, blue colours indicate agreement between the two years (positive MESS values), whereas red colours (negative MESS values) indicate dissimilarities.

**Fig. S4.2.** MESS analysis between 2000 and 2010 for an alternative set of more temporarily stable climate variable. In the MESS maps, blue colours indicate agreement between the two years (positive MESS values), whereas red colours (negative MESS values) indicate dissimilarities.

| Species | AUC – Bio15 | AUC – Bio3 |
| --- | --- | --- |
| AARV | 0,69 | 0,75 |
| CBRA | 0,56 | 0,6 |
| CCAN | 0,52 | 0,53 |
| CCANN | 0,6 | 0,54 |
| CCHL | 0,65 | 0,71 |
| CPAL | 0,61 | 0,6 |
| ECIA | 0,6 | 0,61 |
| ERUB | 0,57 | 0,53 |
| FCOE | 0,54 | 0,52 |
| GGLA | 0,6 | 0,64 |
| LCOL | 0,72 | 0,77 |
| OORI | 0,53 | 0,56 |
| PATE | 0,51 | 0,51 |
| PCRI | 0,55 | 0,53 |
| PIBE | 0,51 | 0,58 |
| PMAJ | 0,65 | 0,55 |
| PMOD | 0,7 | 0,68 |
| PVIR | 0,57 | 0,62 |
| RIGN | 0,59 | 0,59 |
| SATR | 0,5 | 0,54 |
| SCOM | 0,67 | 0,64 |
| SSER | 0,5 | 0,53 |
| STOR | 0,63 | 0,62 |
| STUR | 0,63 | 0,55 |
| SUND | 0,52 | 0,51 |
| TMER | 0,63 | 0,5 |
| TTRO | 0,65 | 0,62 |

**Table S4.1.** AUC values for climate models calibrated using Bio5, Bio12 and Bio15; and Bio 3, Bio5 and Bio12.


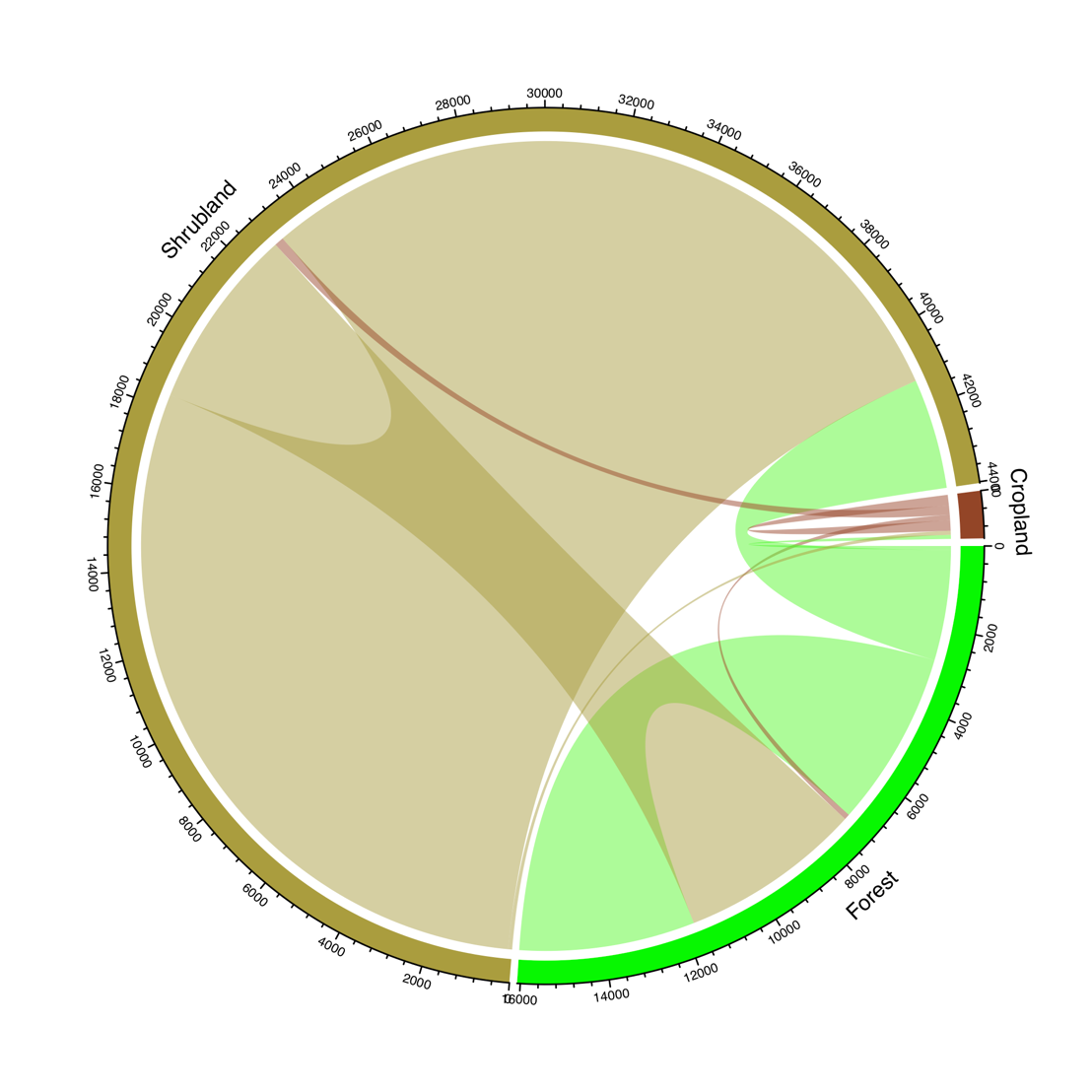


**Fig. S4.3.** Circular plot illustrating the land cover type transitions between 2000 and 2010, in hectares (ha) The size of the lines is proportional in width to the contribution of each land cover type to the change. The colours refer to the land cover types.

**Fig. S4.4**. Area affected by wildfires (in red) between 2001 and 2010.

**References**

1. Moulds, S. lulcc: Land Use Change Modelling in R. R package version 1.0.2. https://cran.r-project.org/package=lulcc. (2017). at <https://cran.r-project.org/package=lulcc>

2. R Core Team. *A language and environment for statistical computing.* (R Foundation for Statistical Computing, Vienna, Austria., 2015). at <http://www.r-project.org/.>

3. Gu, Z. Circlize implements and enhances circular visualization in R. *Bioinformatics* **30,** 2811–2812 (2014).

4. Regos, A., Ninyerola, M., Moré, G. & Pons, X. Linking land cover dynamics with driving forces in mountain landscape of the Northwestern Iberian Peninsula. *Int. J. Appl. Earth Obs. Geoinf.* **38,** 1–14 (2015).

5. Regos, A. *et al.* Rural abandoned landscapes and bird assemblages: winners and losers in the rewilding of a marginal mountain area (NW Spain). *Reg. Environ. Chang.* **16,** 199–211 (2016).
